# Death of tonsillar B cells by NETosis

**DOI:** 10.1038/s41420-023-01402-4

**Published:** 2023-03-30

**Authors:** Ameera Bukhari, Olga Kalinina, Katherine L. Knight

**Affiliations:** Department of Microbiology and Immunology, Loyola University Chicago, 2160 S. First Ave, Maywood, IL 60153 USA

**Keywords:** Immune cell death, Cell death

## Abstract

Regulating B cell death is essential for generating antibodies and maintaining immune tolerance. B cells can die by apoptosis, and we report that human tonsil B cells, but not peripheral blood B cells also die by NETosis. This cell death is density-dependent, characterized by the loss of cell and nuclear membrane integrity, release of reactive oxygen species, and chromatin decondensation. Tonsil B cells secrete high levels of TNF, and inhibiting TNF prevented chromatin decondensation. By in situ fluorescence microscopy, B cell NETosis, as identified by the hyper citrullination of Histone-3, was localized to the light zone (LZ) of germinal centers in normal tonsil and overlapped with the B cell marker CD19/IgM. We propose a model in which stimulation of B cells in the LZ induces NETosis, driven in part by TNF. We also provide evidence that NETosis of tonsil B cells may be inhibited by an unidentified factor in tonsil. The results describe a previously unidentified form of B cell death and suggest a new mechanism to maintain B cell homeostasis during immune responses.

## Introduction

B cell death is key to eliminating self-reactivity during lymphopoiesis and in maintaining homeostasis during and after antigen-induced immune responses. Impaired B cell death due to genetic or environmental causes can lead to serious outcomes such as autoimmunity and lymphomas. Although B cells are known to die by apoptosis [1], in 1896, Ferdinand Gumprecht described an unusual morphology of B cells in the blood of chronic lymphocytic leukemia patients [2, 3]. The morphology of these cells, known as smudge cells or Gumprecht shadows, suggests that they may not be dying by apoptosis. Instead, unlike apoptosis, which is characterized by the shrinkage and fragmentation of dying cells into apoptotic bodies, the smudge morphology is characterized by a lack of defining structural cell elements such as nuclear and cell membrane, and the cells appear as a blur or a shadow on blood smear slides [4, 5]. The smudge morphology was considered an important biological phenomenon, especially in patients with CLL, where the presence of these cells and their frequency have been used as a diagnostic tool, as well as a prognostic marker to predict the 10-year survival rate of these patients [5–7]. The origin and significance of smudge cells have not been investigated extensively. Although some investigators considered the smudge morphology an artifact of the slide preparation [8, 9], others explain this morphology as due to the fragile nature of cancer cells [4, 10, 11]. Currently, the biological significance of smudge cells remains elusive.

B cells with the smudge morphology have a swollen nucleus with splattered chromatin described as a “basket”, sometimes referred to as “basket cells” [3]. These cells are likely dead or dying, and of the >12 death pathways known for cells, the swollen nucleus morphology is found only in neutrophil extracellular trap formation (NETosis) [12]. Cell death by NETosis is also characterized by the loss of nuclear and cell membrane integrity, which is followed by decondensation and release of chromatin [13]. We surmised that smudge cells likely represent B cells dying by NETosis, and we investigated if normal B cells die in a similar manner. We examined B cells in peripheral blood and in the tonsil, and found that B cells in tonsil, but not in peripheral blood undergo NETosis, and further, that this phenomenon appears to occur in vivo, primarily in germinal centers.

## Results

### Spontaneous death of tonsillar B cells

**Fig. 1:**
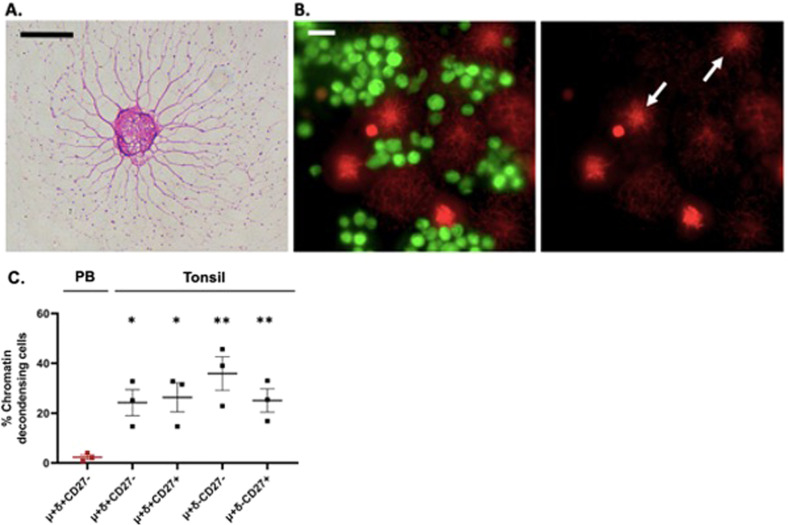
Spontaneous death of tonsillar B cells. **A** Wright-Giemsa stain of human tonsil IgM+ IgD+ CD27- B cell after 1 h at 37 °C. Scale bar = 10 µm. **B** Tonsillar IgM+ IgD+ CD27- B cells stained with Calcein AM (green) and PI (red); PI-only shown in right panel. Scale bar = 10 µm. **C** Percent PB and tonsil B cells with chromatin decondensation after one 1 h culture. Tonsil B cell populations compared to PB. Each point represents a different sample; paired *t*-test, *P-value < 0.05, **P-value < 0.01.

During a routine examination of B cells in culture, we observed that many human tonsillar B cells exhibited an unusual star-like shape with a shredded nuclear appearance (Fig. 1A). To further investigate these cells, we isolated mature IgM^+^ B cells by negative selection, from peripheral blood and tonsil, and after a short incubation (1–3 h) at 37 °C in cell culture plates or on glass slides, the cells were fixed and stained with Wright-Giemsa stain. Whereas we found that 10 to 50% of the tonsil B cells exhibited this unusual appearance, peripheral blood B cells had normal morphology with compact nuclei and tight cytoplasm (data not shown). To test if the unusual appearance of tonsil B cells was due simply to cell membrane extensions (i.e. tunneling nano tubes) in live cells, or to DNA decondensation in dead cells, we stained the cells with Calcein AM to detect live cells and cultured them in the presence of propidium iodide (PI) to detect extracellular double stranded DNA in dead cells. All star-like cells stained with PI and not with Calcein AM, showing that this unusual morphology corresponds to unwound nuclear chromatin, i.e., chromatin decondensation, and cell death. This cell death is distinct from apoptosis, as the cells do not show evidence of nuclear shrinkage nor apoptotic bodies (Fig. 1B).

We tested if all subpopulations of tonsil B cells undergo cell death with similar morphology by sorting naïve (IgM^+^ IgD^+^ CD27^−^, IgM^+^ IgD^−^ CD27^−^) and memory (IgM^+^ IgD^+^ CD27^+^, IgM^+^ IgD^−^ CD27^+^) B cells, plating them as above, and staining with Wright-Giemsa stain. We found that 10–50% of the cells in each of the four subpopulations developed the star-like morphology, indicating that they had undergone similar spontaneous cell death (Fig. 1C). These findings suggest that normal tonsillar naïve and memory B cells can undergo death by a pathway other than apoptosis.

### Netosis of tonsil B cells

**Fig. 2:**
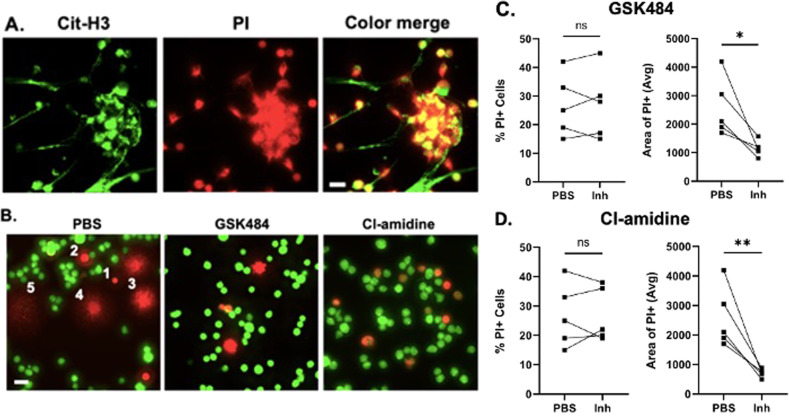
NETosis-like death of tonsillar B cells. **A** Immunofluorescence of tonsillar B cells stained for hyper-Cit-H3 and DNA. Tonsillar IgM+IgD+CD27- B cells stained with anti-hyper-Cit-H3 (green) and PI (red) after 2 h at 37 °C. Scale bar = 10 µm. Data are representative of 4 subjects. **B** Inhibition of chromatin decondensation by PAD inhibitors. Tonsil IgM+IgD+CD27- B cells treated with PAD inhibitors, GSK484 or Cl-amidine. Cells labeled with Calcein AM (green); dead cells labeled with PI (red). Photos are representative of 5 subjects. Numbers 1–5 represent putative chromatin decondensation steps ((1) compact nucleus; (2) and (3) swollen nucleus; (4) swollen nucleus with fine DNA branches; (5) fine strands of DNA). **C**, **D** Quantification of cell death (PI+) and chromatin decondensation (average area of PI + cells (square pixel)) in untreated and GSK484- (**C**) or Cl-amidine- (**D**) treated cells; paired *t*-test, *P-value < 0.05, **P-value < 0.01.

Mammalian cells die by one of more than twelve death pathways. The chromatin decondensation that we observed in dying tonsillar B cells (Fig. 1) is reported only for death by nuclear extracellular trap formation (NETosis) [12]. To assess if these cells are dying by NETosis, we tested if the unwound DNA is associated with the release of reactive oxygen species (ROS) in culture, similar to neutrophil NETosis [14, 15]. ROS production in tonsil B cells was detected within 30–45 min of culture and its signal was associated with the unwound DNA (Supplementary Fig. 1). The chromatin decondensation during NETosis occurs through the citrullination of arginine residues in DNA-binding histones, which neutralizes the positive charge on histones and reduces their capacity to bind negatively charged DNA [16–18]. Histone citrullination results in unwound chromatin, and we asked if histones in B cells are citrullinated, indicating that they are dying by NETosis. We plated tonsil mature naïve B cells on glass slides for 2 h and after staining with anti-hyper-Cit-H3 antibody (Ab) and propidium iodide (PI), we found the staining of hyper-Cit-H3 overlapped with the unwound chromatin PI signal (Fig. 2A), suggesting that the histone of dying cells was hyper-citrullinated. Almost no hyper-Cit-H3 was detected in living tonsillar B cells (Supplementary Fig. 2).

Histone citrullination requires the action of peptidyl arginine deiminases (PADs) [17], and to confirm that the DNA decondensation required histone citrullination, we cultured the mature naïve tonsil B cells with the PAD4 inhibitor, GSK484, and Cl-amidine, a Pan-PAD inhibitor. Treatment with these inhibitors reduced the DNA-decondensation significantly, as observed by the decrease in the size and star-like projections of PI^+^ cells, but they had little or no effect on cell survival, as seen by similar % PI^+^ B cells with and without inhibitors (Fig. 2B–D). We conclude that the DNA decondensation occurs through histone citrullination, confirming that the cell death occurs by NETosis.

### Enhancement of tonsillar B cell NETosis by TNF

**Fig. 3:**
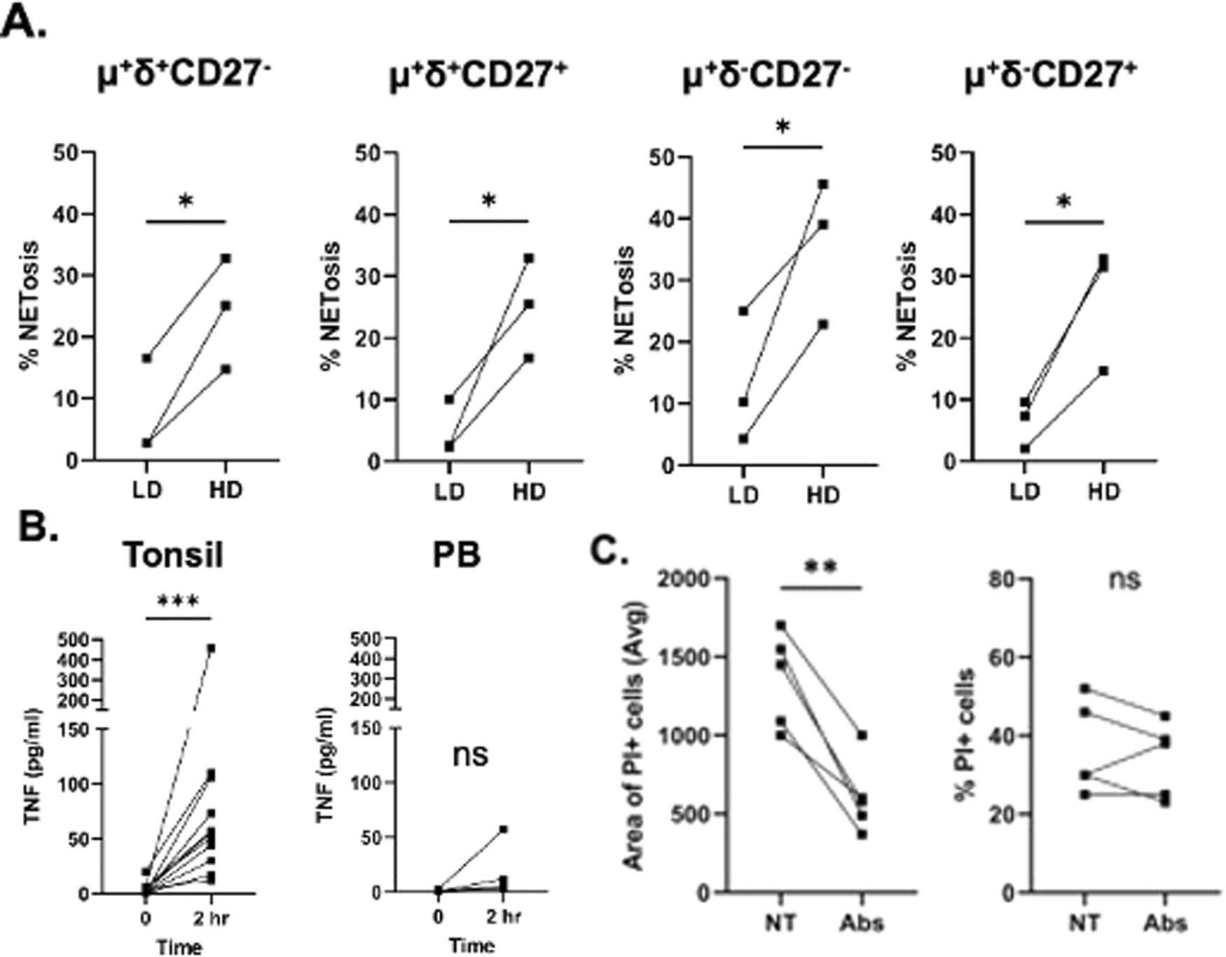
Effect of cell density and secreted TNF on B cell death by NETosis. **A** Cells cultured at low density (LD) (5,000 cell/cm^2^) versus high density (HD) (50,000 cell/cm^2^). Paired *t*-test; *P < 0.05. **B** TNF secretion by tonsil and PB IgM^+^ IgD^+^ CD27^−^ B cells. TNF secretion in culture supernatant from cells killed abruptly by freezing or after 2 h incubation at 37 °C. Tonsil *N* = 11. T_0_ = 2.5 pg/ml (median); 2 h culture = 56 pg/ml (median). PB *N* = 4. T_0_ = 1.3 pg/ml (median); 2 h culture = 8.7 pg/ml (median). Wilcoxon test, Tonsil ***P < 0.001, PB *P* = 0.12. **C** Effect of TNF neutralizing Abs on B cell NETosis (left) and cell survival (right); Paired *t*-test, **P = 0.002.

The experiments described above were performed with B cells plated at 50,000–100,000 cells/cm^2^. Significantly less NETosis was found in cells plated at lower cell density (5000 cell/cm^2^) (Fig. 3A). We hypothesized that at high density, tonsillar B cells secrete a molecule that enhances NETosis. Neutrophil NETosis can be triggered in sterile conditions by cytokines such as IL1-β and TNF [19], and after 2 h culture of tonsillar mature naïve B cells at high density, while we found no IL-1β (<1 pg/ml) in the supernatant, we found high levels of TNF (median = 56 pg/ml) (Fig. 3B). Nearly all of this TNF was secreted during the 2 h culture because <2.5 pg/ml was in the supernatant of cells abruptly killed by flash freezing at the start of the culture. The concentration of TNF in culture increased with increased number of cells/well in culture (Supplementary Fig. 3A), which might explain the high rate of B cell NETosis at high cell density. However, when we normalized the amount of TNF production to the number of cultured cells (Supplementary Fig. 3B), there was no significant difference between the different cell densities, which indicates that the high cell density does not increase TNF production. In the presence of TNF, and TNF-R1/2 neutralizing Abs, the number of B cells undergoing NETosis (as identified as large DNA unwinding, PI^+^ cells), was significantly reduced (Fig. 3C and Supplementary Fig. 4), although the number of dead cells (PI^+^) did not change. These data are consistent with previous reports that TNF enhances protein citrullination but does not initiate cell death [19–21]. The data indicate that in B cells dying by NETosis, TNF functions downstream on an as-yet-unknown cell death trigger.

### NETosis of B cells in tonsillar tissue

**Fig. 4:**
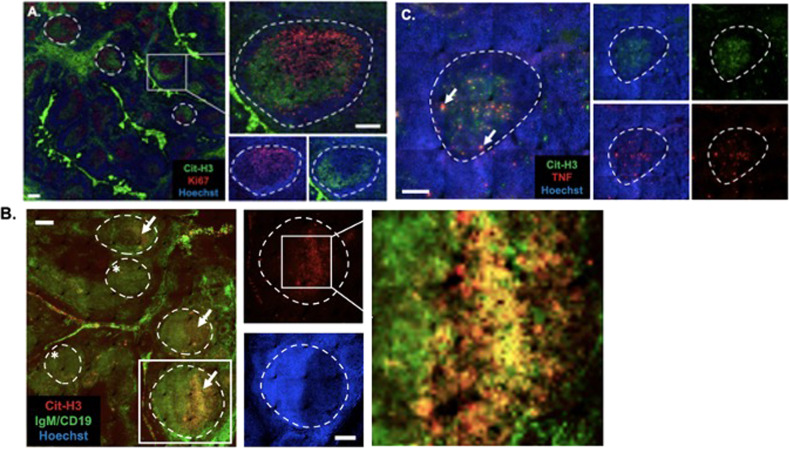
Hyper-Cit-H3 in tonsil tissue. Immunofluorescence on cross sections of human tonsil. Scale bar = 200 μm. **A** Hyper-Cit-H3 and Ki67. B cell follicles highlighted with dashed lines. Square is expanded and shown with single color in lower right. **B** Hyper-Cit-H3 and B cell markers CD19/IgM. Arrows point to areas with high HyperCit-H3 signal (GC). Asterisks indicate primary B cell follicles. Square is shown on right with single stain, Cit-H3 (top) or Hoecht (bottom). Far-right panel shows overlapping hyper-Cit-H3 and IgM/CD19 signal (yellow). **C** Hyper-Cit-H3 and TNF. Arrows show areas with overlapping hyper-Cit-H3 and TNF signal. Right panel shows single color images of Cit-H3 (top) and TNF (bottom) with and without the nuclear stain Hoechst. Data are representative of 4 subjects.

We assessed B cell NETosis in situ by staining tonsillar tissue sections with anti-hyper-Cit-H3 and with anti-Ki67 Ab, which identifies proliferating B cells in the dark zone (DZ) of germinal centers (GC) [22]. Cells stained with anti-hyper-Cit-H3 Ab were found in the GC light zone (LZ) (Ki67^−^), but not in the DZ (Fig. 4A). To confirm that the hyper-Cit-H3 staining was associated with B cell follicles, we used the B cell markers CD19 and IgM, and found the hyper-Cit-H3 signal substantially colocalized with IgM and CD19 in the GCs (Fig. 4B); no hyper-Cit-H3 staining was found in primary follicles where B cells are in a resting state. In situ analysis of spleen sections showed that hyper-Cit-H3 was found in B cell-rich areas, indicating that B cell NETosis is likely occurring in other secondary lymphoid tissues (Supplementary Fig. 5). Additional studies are needed to confirm the identity of hyper-Cit-H3 positive cells.

Because TNF enhances B cell NETosis ex vivo, we tested if TNF colocalized with hyper-Cit-H3 in GCs. As expected, TNF was localized to GCs [23], and it colocalized with hyper-Cit-H3 (Fig. 4C). We conclude that some B cells are undergoing NETosis in the LZ of GCs, and we suggest that this is likely fostered by high levels of TNF.

**Fig. 5:**
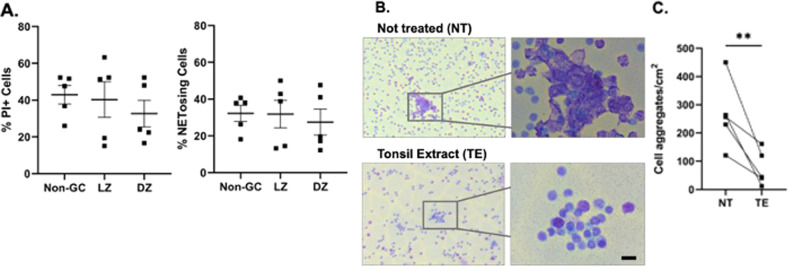
Ex vivo NETosis of non-GC versus GC B cells. **A** Sorted Non-GC, light and dark zone B cells after 1 h culture. Percentage of total dead cells (left) and NETosing B cells (right). For statistical analysis, LZ and DZ B cells were compared to non-GC B cells; paired *t*-test, differences are not significant. **B**, **C** Tonsil IgM^+^ IgD^+^ CD27^−^ were isolated and cultured in FCS or tonsil extract (TE). **B** Representative photo of untreated (UT) (top) or cells treated with tonsil extract (TE) (bottom). **C** Quantification of cell aggregates/cm^2^ in cells cultured in FCS or TE; Paired *t*-test, **P-value < 0.01.

Based on the in situ data, we hypothesized that LZ B cells undergo NETosis-like death significantly more than non-GC or DZ B cells. We tested this by sorting GC LZ, DZ, and non-GC B cells from tonsil (Supplementary Fig. 6) and after plating on glass slides to visualize cells undergoing NETosis, we found that similar numbers of B cells in all groups died by NETosis (Fig. 5A). These data led us to test if NETosis-like death of B cells is a default state for tonsil B cells unless they are rescued, potentially by a survival signal present in the tissue. Incubation of tonsil B cells with extract from tonsillar tissue significantly decreased B cell NETosis (Fig. 5B, C), indicating that the tissue contains a molecule(s) that can rescue B cells from NETosis-like death.

## Discussion

We show that B cells in tonsil but not in peripheral blood die by NETosis, the process by which neutrophils generally die [24]. B cell NETosis is associated with the production of high levels of reactive oxygen species (ROS) and appears to proceed through the same steps described for neutrophil NETosis, chromatin swelling and DNA decondensation, until only fine strands of DNA are observed (steps 1–5 in Fig. 2B—left panel) [13, 25]. The chromatin decondensation and the DNA release require histone citrullination, and as expected, NETosis of tonsil B cells was inhibited by the PAN-PADs inhibitors, Cl-amidine and GSK484, both of which inhibit histone citrullination. We searched for signs of chromatin decondensation and DNA release in tonsil tissue by staining with Wright-Giemsa and Hoechst 33342, however identifying fine DNA structures as evidence of NETosis in tissue was technically challenging due to the high density of cells. The absence of naked DNA in tissue might also be due to its rapid clearance by tissue clearing mechanisms such as by macrophages, or to the action of DNases. For these reasons, we used Ab to Cit-H3 as a marker for NETosis both in situ and in vitro. We recognize that citrullinated histones can also facilitate gene transcription. We think, however, that the citrullination signal in our experiments reflects cell death rather than gene transcription because we used an Ab specific for hyper-citrullination of histone 3 (citrulline R2 + R8 + R17), which so far, has been associated only with NETosis, which, to our knowledge, remains the most credible and widely used marker of NETosis in neutrophils and in other types of immune cells [16, 26–31].

The NETosis signal observed in situ appears to be localized primarily to GC-LZ, an area in which B cells are known to die [22, 32]. The NETosis signal co-localized with the B cell markers, IgM and CD19, and no significant NETosis was found in primary B cell follicles or in the GC-LZ. We suggest that in the GC-LZ, some B cells are eliminated by NETosis due to autoreactivity, or to the cells’ inability to participate in selective processes, including antigen uptake and interaction with Tfh cells. We cannot, however, rule out the possibility that some of the NETosis signal in GC-LZ is due to another cell type.

The level of Cit-H3 signal in the tonsil tissue was high, raising the question about the extent of NETosis in the tissue. Based on our in vitro data, we think that the high Cit-H3 signal does not reflect the number of dying cells in a linear fashion because the in vitro data show that when B cells die by NETosis, the chromatin expands, and cells come to occupy 10–20X the area of a living lymphocyte (Fig. 1B).

How is it that tonsil B cells, but not PB B cells undergo NETosis? Rocha Arreta et al. [33] reported that activation of PB B cells by PMA, ionomycin, anti-IgM and LPS, or serum from patients with SLE, triggered the release of extracellular DNA. We suggest that in tonsil, unlike in PB, B cells are frequently exposed to bacteria and bacterial TLR agonists, which induce a NETosis-like reaction.

When we measured the rate of NETosis in the GC-DZ and LZ B cells, the data from both groups clustered into two subgroups, one with a higher rate of NETosis than the other (Fig. 5A). This intra-group difference is likely due to immune responses that occurred in the tissue before the isolation of these cells, which leads to the high susceptibility to NETosis in some individuals. Although these tissues were evaluated as “normal” by pathology, we cannot rule out the possibility that these cells were actively participating in an immune response against a pathogen, especially likely because the cells were isolated as activated B cells (CD38^+^).

The finding that, in situ, the cells undergoing NETosis appear localized to GC LZ, and yet in vitro, GC DZ B cells and as many as 50% of other B cells (non-GC) undergo NETosis, suggests that tonsil tissue likely contains a molecule that inhibits NETosis of B cells in situ outside the GC LZ. Indeed, we found that extracts of tonsil tissue prevent NETosis of tonsil B cells in vitro. We supplemented tonsil B cells in vitro with B cell survival molecules, recombinant Baff and April, but could not rescue the B cells from NETosis in our culture system (data not shown). This survival factor in tonsil is yet to be identified. It is also noteworthy that tonsil B cells, unlike PB B cells secrete large amounts of TNF, which enhances the DNA decondensation step of NETosis. The extent to which the high level of TNF in tonsil contributes to NETosis in GC LZ also remains a mystery.

B cells undergoing NETosis morphologically appear highly similar to smudge cells with undefined nuclear and cell membranes found in CLL patients’ blood. We propose that these smudge cells are leukemic B cells dying by NETosis. Considering our results that PB B cells from healthy individuals do not undergo NETosis, we suggest that the smudge cells in PB of CLL patients is due to the activation status of these cells. In contrast to PB B cells from healthy individuals, CLL B cells express multiple activation markers, including CD38, CD23, CD25, CD69, and CD71 [34, 35], and stimulation of PB lymphocytes with TLR agonists induces NETosis-like death of B cells [33]. The CLL patients also have high levels of serum TNF, much of it coming from lymphocytes [36–38], and patients undergoing anti-TNF treatment, such as for rheumatoid arthritis and inflammatory bowel disease have a significantly higher risk of developing lymphomas than normal individuals [39–42]. These findings suggest that TNF not only contributes to the NETosis death pathway, but also is an important cytokine in the CLL disease process. High levels of smudge cells in CLL patients correlates with increased life span of the patients [4], leading us to suggest that B cell NETosis offers some protection by killing activated cells.

**Fig. 6:**
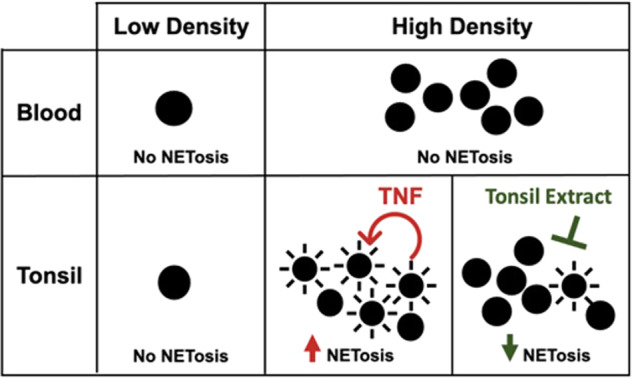
Model of NETosis of tonsillar B cells. Tonsil but not PB B cells undergo NETosis at high cell density. The chromatin decondensation in B cell NETosis is enhanced by B cell secretion of TNF. Treatment of B cells with tonsil tissue extract inhibits NETosis of tonsillar B cells.

Based on our in vitro and in situ data, we propose a model in which B cells in GCs, but not in primary B cell follicles, receive an unidentified signal that triggers their NETosis in the presence of TNF (Fig. 6). Our data show that LZ and DZ B cells undergo comparable levels of NETosis in vitro but our in situ data suggest that cells in the DZ are protected from NETosis in the tissue. We propose the presence of a survival signal(s) that antagonizes the trigger for NETosis in GC DZ, thereby rescuing the cells from death. This signal is likely absent when the cells are released from the tissue, rendering them vulnerable to NETosis as the default death pathway for tissue B cells after stimulation.

In summary, we report that human tonsil B cells, but not PB B cells die by NETosis. These B cells secrete high levels of TNF, which contributes to the NETosis process. In situ, NETosis of B cells, as evidenced by the presence of citrullinated histone, was limited to the GC LZ. Soluble factor(s) in the tissue were shown to rescue the cells from undergoing this death pathway. The biologic and immunologic significance of B cell NETosis in the GC LZ, as well as the mechanisms driving this NETosis are yet to be fully understood, both in health and in B cell disease.

## Materials and methods

### Human specimens—collection and preparation

Histologically normal spleen paraffin-embedded tissue blocks and fresh tonsil samples were obtained from the Department of Pathology at Loyola University Chicago. Tonsil donors’ age ranged from 2 to 30 years (mean = 12 years; median = 9.5 years); male: female = 1:1. Tonsil mononuclear cells were teased from fresh 1–3 cm^3^ tissue pieces and passed through 100 µm cell strainer. Peripheral blood was obtained from healthy adult donors and mononuclear cells were isolated from buffy coats. Lymphocyte separation medium (Corning) was used for additional lymphocyte purification. Tonsils were embedded in OCT for histological studies. The use of human specimens was approved by the institutional review board at Loyola University Chicago.

### Isolation and culture of B cell subsets

Mononuclear cells isolated from tonsil tissue were treated with FcR block (Biolegend—Cat: 422302) before staining with fluorescent antibodies: anti-CD19 (HIB19), anti-IgM (MHM-88), anti-IgD (IA6-2), anti-CD27 (LG.3A10). Dead cells were excluded with fixable cell viability dye (eBioscience—eFluor 780). Naïve and memory B cells were analyzed and sorted using FACSAria III. Negative selection of B cells was performed with StemCell—EasySep B cell isolation kit (Cat: 17954) and shown by flow cytometry to be >98% pure. B cells were counted and plated on glass slides (Fisherbrand—Superfrost Plus microscope slides) or glass coverslips at high density (≥5 × 10^4^ cell/cm^2^) or low density (5 × 10^3^ cell/cm^2^). The cells were cultured in FCS to facilitate imaging and were incubated in humidified chambers at 37 °C and 5% CO_2_ for 1–3 h. The cells were fixed with 100% methanol or 4% paraformaldehyde and stained with Wright- Giemsa or immunofluorescence for analysis by microscopy.

### Microscopy

Bright field images were acquired with a Leica DM IRB microscope (Leica Microsystems) using a MagnaFire 2.1 C digital camera system (Optronics). For fluorescence microscopy, cells and tissues were examined with a DeltaVision widefield fluorescence microscope (Applied Precision) equipped with a digital camera (CoolSNAP HQ; Photometrics).

### Detection of reactive oxygen species (ROS)

Negatively selected B cells were stained with Calcein AM (Millipore Sigma—Cat: 17783), cultured on glass slides and incubated in a humidified chamber at 37 °C and 5% CO_2_ for 45 min–1 h. CellROX (Invitrogen–Cat: C10422) was used according to manufacturer protocol to detect reactive oxygen species. Cells were fixed with 4% paraformaldehyde for analysis by microscopy.

### Cytokine quantitation

Negatively selected B cells (5 × 10^4^ cell/well) were cultured in 50 µl RPMI supplemented with 10% FCS in 96-well cell culture plates at 37 °C and 5% CO_2_ and supernatant was collected after 2 h for cytokine analysis using a cytometric bead array kit (Biolegend–Cat: 740809). As negative control, cytokines in supernatant of flash-frozen cells were measured. Data were analyzed using LEGENDplex data analysis software.

### PADs inhibition and TNF and TNF-R1/2 neutralization

Negatively selected B cells were stained with Calcein AM (Millipore Sigma—Cat: 17783) and incubated on ice for 1 h with: GSK484 (500 nM), Cl-amidine (20μM), 1μg/ml of neutralizing anti-TNF (MAb1), anti-TNF-R1 (MAB225R), and anti-TNF-R2 (MAB226) or mouse IgG1 isotype control (MOPC-21). Cells were plated on glass slides in the presence of propidium iodide (PI) (Millipore Sigma—Cat: P4170) and incubated for 1 h at 37 °C and 5% CO_2_ and fixed with 4% paraformaldehyde for analysis by microscopy.

### Immunofluorescence for cells and tissue sections

Cells and tissue sections were fixed with 4% paraformaldehyde, permeabilized with 0.1% triton X-100, and incubated with FcR block (Biolegend—Cat: 422302) and endogenous peroxidase block (Vector laboratories—Cat: SP-1000-600). The cells were stained with the following FITC- or biotin-labeled primary antibodies: anti-CD19 (HIB19), anti-IgM (MHM-88), anti-Ki67 (SolA15), anti-Cit-H3 (ab5103), anti-TNF (MAb11). HRP-conjugated anti-FITC (AB_2314402) and anti-biotin (AB_2339006) antibodies were used with tyramide signal amplification reagent (Thermo Fisher – Cat: B40953, B40957) to detect secondary antibodies.

### Quantitation of NETosis from bright field and immunofluorescence images

In bright field images, cells were stained with Wright-Giemsa stain and NETosis was quantified by counting cell aggregates with disrupted nuclear membrane and unwound chromatin in the entire 1 cm^2^ culture area on the culture glass slide. Slides was evaluated by four blinded technologists and all results differed by less the 5%. For fluorescent images, cells were stained with Calcein AM (Millipore Sigma—Cat: 17783) and cultured in the presence of propidium iodide (PI) (Millipore Sigma—Cat: P4170) or stained for Cit-H3 (ab5103). Cells were fixed and we photographed 10 random fields (each field contained ≥100 cells) and counted propidium iodide positive (PI^+^) cells or Cit-H3 positive cells (red) and Calcein AM positive cells (green). Total dead cells were calculated as percent PI^+^ cells of total cells. Average area of PI^+^ cells was used to determine the number of cells undergoing chromatin decondensation: NETosing cells were defined as PI^+^ cells with an area of ≥1000 square pixel (px^2^). All calculations were performed using ImageJ (U. S. National Institutes of Health, Bethesda, Maryland).

### Preparation of tonsil extract

We induced a single cut in a histologically normal tonsil and washed inside the tissue multiple times using FCS (1 ml/gm tissue). Cell-free supernatant was collected and sterilized with 20 µm filter and used to test for NETosis-inhibition activity.

### Statistical analysis

Statistical significance was determined by paired or unpaired Student’s *t*-test using GraphPad Prism software (La Jolla, CA).

## Supplementary information

Supplementary figure 1

Supplementary figure 2

Supplementary figure 3

Supplementary figure 4

Supplementary figure 5

Supplementary figure 6

## Data Availability

The data generated during and/or analysed during the current study are available from the corresponding author on reasonable request.
