# Supplementary figures and images for: Death of tonsillar B cells by NETosis
Source: Cell Death Discov. 2023 Mar 30;9:108. doi: 10.1038/s41420-023-01402-4 (PMC10063674; doi:10.1038/s41420-023-01402-4)

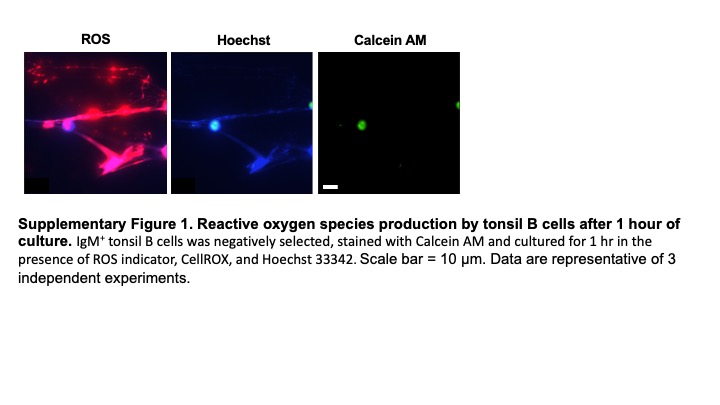

Supplement: Supplementary file 1 — Supplementary figure 1 [file 41420_2023_1402_MOESM1_ESM.jpg]

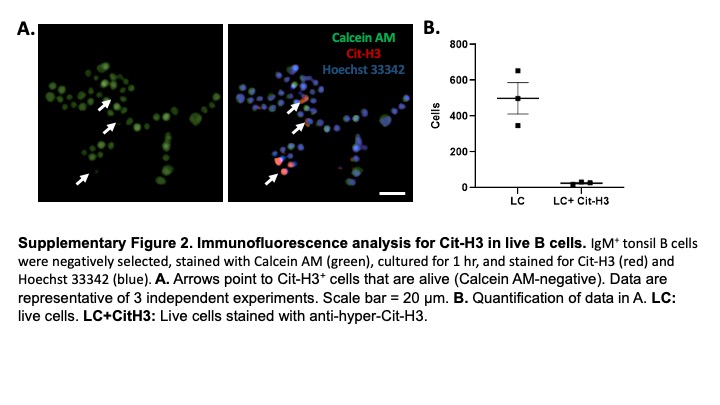

Supplement: Supplementary file 2 — Supplementary figure 2 [file 41420_2023_1402_MOESM2_ESM.jpg]

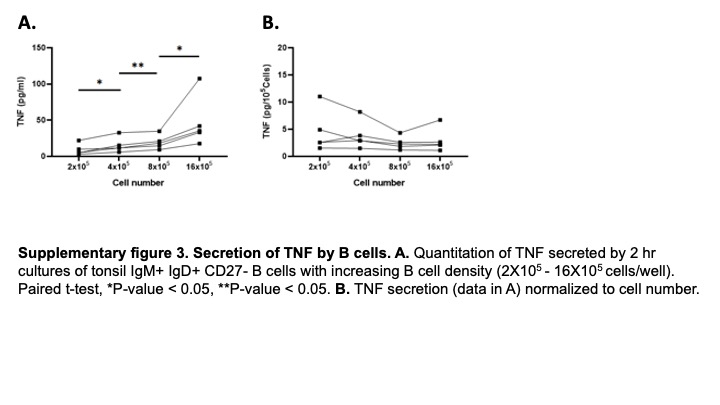

Supplement: Supplementary file 3 — Supplementary figure 3 [file 41420_2023_1402_MOESM3_ESM.jpg]

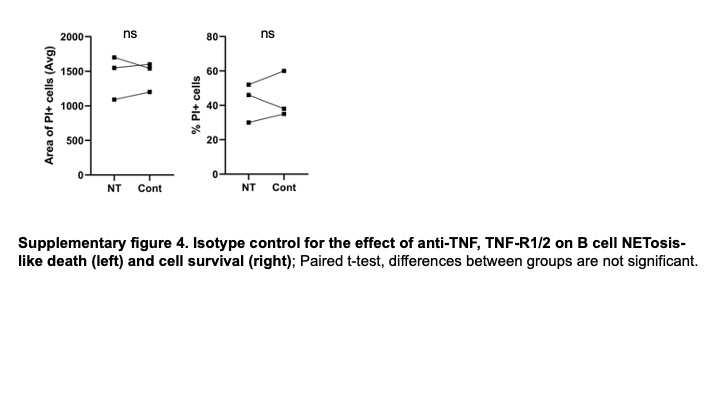

Supplement: Supplementary file 4 — Supplementary figure 4 [file 41420_2023_1402_MOESM4_ESM.jpg]

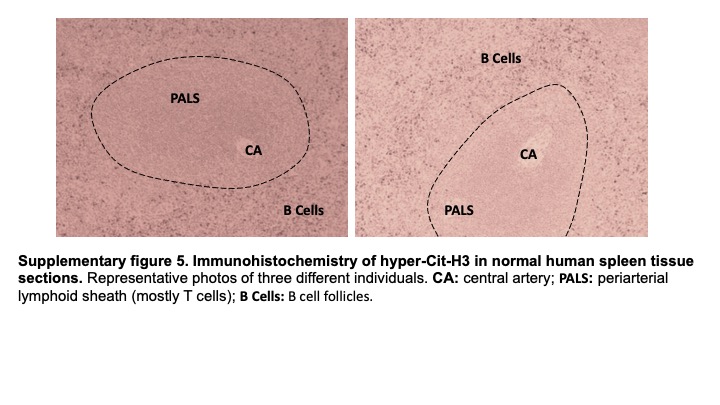

Supplement: Supplementary file 5 — Supplementary figure 5 [file 41420_2023_1402_MOESM5_ESM.jpg]

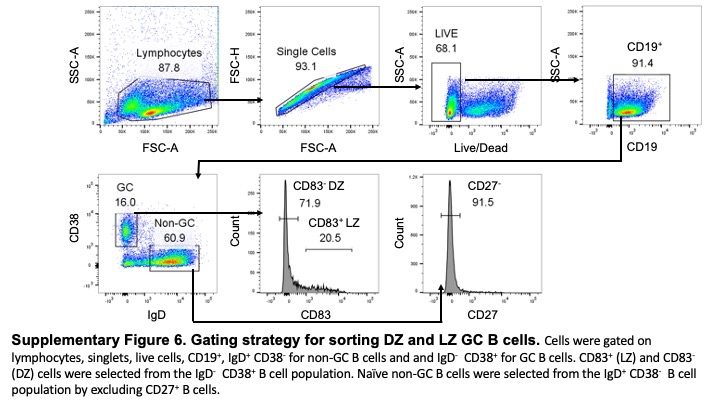

Supplement: Supplementary file 6 — Supplementary figure 6 [file 41420_2023_1402_MOESM6_ESM.jpg]
